# Evaluation of Pre-Analytical Variables for Human Papillomavirus Primary Screening from Self-Collected Vaginal Swabs

**DOI:** 10.1016/j.jmoldx.2024.02.006

**Published:** 2024-06

**Authors:** Michelle Qi, Anissa R. Naranjo, Abigail J. Duque, Thomas S. Lorey, Jeffrey M. Schapiro, Betty J. Suh-Burgmann, Michael Rummel, Stephen J. Salipante, Nicolas Wentzensen, Dina N. Greene

**Affiliations:** ∗ LetsGetChecked Laboratories, Monrovia, California; † The Permanente Medical Group, Northern California Kaiser Permanente Regional Reference Laboratory, Oakland, California; ‡ Department of Research, The Permanente Medical Group, Oakland, California; § Department of Laboratory Medicine and Pathology, University of Washington, Seattle, Washington; ¶ Division of Cancer Epidemiology and Genetics, National Cancer Institute, Bethesda, Maryland

## Abstract

Human papillomavirus (HPV) primary screening is an effective approach to assessing cervical cancer risk. Self-collected vaginal swabs can expand testing access, but the data defining analytical performance criteria necessary for adoption of self-collected specimens are limited, especially for those occurring outside the clinic, where the swab remains dry during transport. Here, we evaluated the performance of self-collected vaginal swabs for HPV detection using the Cobas 6800. There was insignificant variability between swabs self-collected by the same individual (*n* = 15 participants collecting 5 swabs per participant), measured by amplification of HPV and human β-globin control DNA. Comparison of self-collected vaginal swab and provider-collected cervical samples (*n* = 144 pairs) proved highly concordant for HPV detection (total agreement = 90.3%; positive percentage agreement = 84.2%). There was no relationship between the number of dry storage days and amplification of HPV (*n* = 68; range, 4 to 41 days). Exposure of self-collected dry swabs to extreme summer and winter temperatures did not affect testing outcomes. A second internal control (RNase P) demonstrated that lack of amplification for β-globin from self-collected specimens was consistent with poor, but not absent, cellularity. These data suggest that self-collected vaginal samples enable accurate clinical HPV testing, and that extended ambient dry storage or exposure to extreme temperatures does not influence HPV detection. Furthermore, lack of β-globin amplification in HPV-negative samples accurately identified participants who required recollection.

Human papillomavirus (HPV) primary screening, defined as HPV detection followed by cytology triage for those with detectable virus, is now considered the most effective and efficient approach to cervical cancer screening in high-resource countries.^1^ This algorithm integrates the greater sensitivity of HPV assays for cervical precancer [cervical intraepithelial neoplasia (CIN) II to III+] with the greater specificity of cytology (Papanicolaou test or CINTech+) for triage of HPV-positive individuals.^2^ Over repeated screening rounds, HPV primary screening affords better or equal protection against cervical cancer compared with cytology alone or cotesting with both HPV and Papanicolaou tests. The high negative predictive value of undetectable HPV affords an extended screening interval and fewer total tests, resulting in a reduction of unnecessary colposcopies and patient anxiety caused by false-positive screening results.^3^

HPV primary screening leverages both the stability of DNA and the sensitivity of targeted nucleic acid amplification assays, eliminating the need for sampling of the cervical transition zone. Basal stem cells at the cervical transformation zone are regarded as the site of carcinogenesis mediated by oncogenic HPV.^4^ Cytologic evaluation allows for detection of the atypical morphologic changes in cervical cells shed from the superficial epithelium that are induced by HPV infection. Because cytology testing depends on well-preserved cellular morphology, direct sampling of the cervix, ideally the cervical transformation zone, is important. Unlike cytology, HPV detection only requires minute quantities of target DNA, eliminating the need for direct sampling of the cervix. Target DNA from oncogenic HPV can be detected in samples from vaginal fluid, urine, and menstrual blood, all amenable to self-collection approaches.^5^, ^6^, ^7^

Numerous comparison studies between self-collected vaginal and provider-collected cervical samples have demonstrated equivalent performance for the detection of CIN II to III+ when a DNA target-amplification assay is used.^8^ Remote self-collection for mail-in HPV testing could greatly expand access, especially for individuals experiencing barriers to office-based screening and/or pelvic examinations. Like home-collect options for colorectal cancer screening, home-collect cervical cancer screening could be supported by telehealth.^9^^,^^10^ Moreover, based on current demographics, it is expected that approximately 90% of individuals undergoing HPV screening will be negative and require no further testing, whereas the 8% to 10% testing positive will require a follow-up pelvic examination for cytology triage.^11^ This could serve to decrease burden on the health care system as it contrasts sharply with conventional testing paradigms, wherein all screened individuals require an office visit and provider collection during the pelvic examination.

Although clinically robust, there are limited data in the literature deriving analytical performance criteria necessary for laboratories to adopt HPV self-collected screening algorithms, particularly for self-collection occurring outside of the clinic. For remote collection, dry swabs are the specimen of choice as they significantly simplify kit design, user experience, and transport. However, validating dry swabs poses an analytical conundrum to the laboratory because unlike wet specimens, there is not a matrix to aliquot and expose to different conditions; once the swab is suspended in buffer, additional pre-analytical challenges to the collection cannot be performed. Here, we evaluated multiple pre-analytical variables to validate the performance of HPV testing performed on self-collected vaginal samples. This assessment included the following: intra-individual variability to understand the fundamental difference between multiple dry swabs collected by the same individual at the same encounter, quantitative comparisons of HPV amplification from provider-collected cervical samples and self-collected vaginal samples, in-transit stability of dry swabs at both ambient and summer/winter conditions, and orthogonal assessment of cellularity among invalid samples. Combined, our goal was to validate the dry swab sample type for molecular HPV testing and to provide a roadmap for other laboratories that are considering a dry-swab collection protocol.

## Materials and Methods

### Study Design and Specimen Collection and Processing

Paired provider/self-collected samples were obtained at Kaiser Permanente Northern California, a large, multicenter integrated health system, from volunteers aged ≥18 years who verbally consented to submit a self-collected vaginal swab at the time of their colposcopy. Volunteers were verbally instructed to insert a Copan (Murrieta, CA) nylon flocked swab into the vagina and to rotate five times before placing the swab into an empty Sarstedt (Newton, NC) 10-mL transport tube. Patient self-collected samples were deidentified. Self-collected test results were not entered into the medical record or used to inform clinical management, and no additional private health information was extracted. As the samples were obtained solely for quality assessment of the self-collected specimens, Kaiser Permanente Institutional Review Board (IRB) approval was not required.

Additional self-collected vaginal swab samples were obtained at LetsGetChecked under an IRB-approved protocol (Advarra IRB Pro00027040, IRB organization number: 0000635; IRB registration number: 00000971; Columbia, MD). Participants at LetsGetChecked were community volunteers aged ≥18 years who signed consent to self-collecting one or more vaginal swabs. For the LetsGetChecked collections, participants were provided with an instructional video.

**Supplemental Figure 1:**
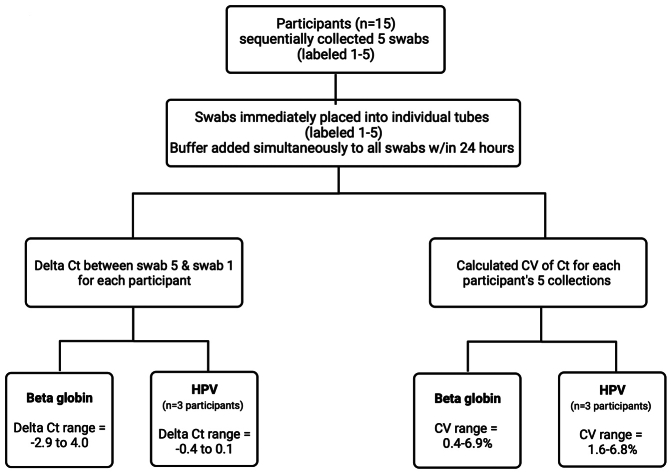
Flow diagram illustrating the experimental process used to evaluate intra-individual swab variability. HPV, human papillomavirus.

**Supplemental Figure 2:**
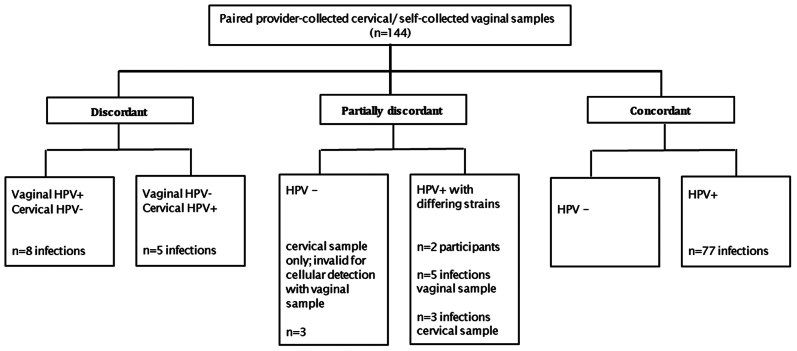
Flow diagram illustrating the categorization of the paired self-collected and provider-collected sample. HPV, human papillomavirus.

**Supplemental Figure 3:**
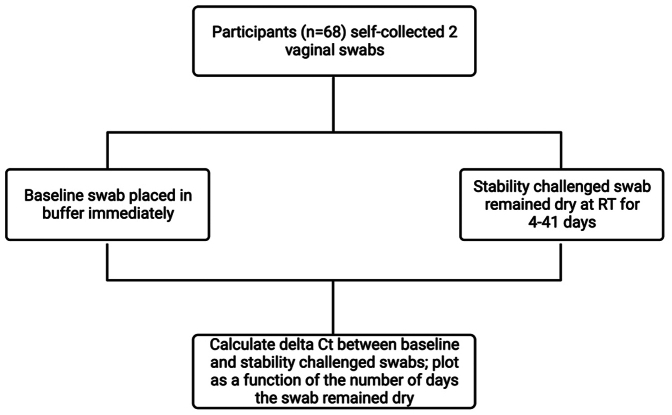
Flow diagram illustrating the experimental process for 30-day ambient temperature stability. RT, room temperature.

**Supplemental Figure 4:**
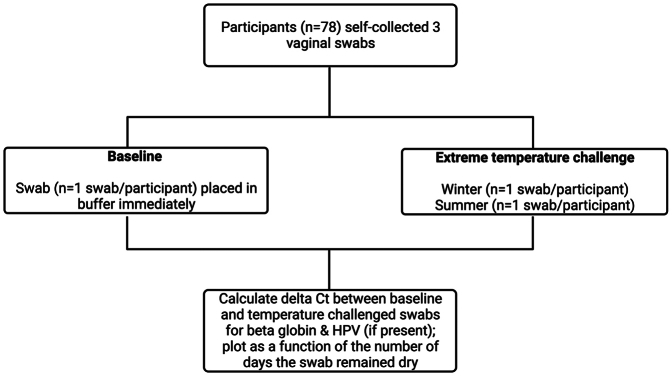
Flow diagram illustrating the experimental process for the summer and winter temperature challenges. HPV, human papillomavirus.

The study design and participants enrolled for various aims of this study are summarized in Supplemental Figures S1–S4.

For all vaginal swab samples, a Copan nylon flocked swab was used for self-collection and then placed into a Sarstedt 10-mL transport tube. At the laboratory, the swab was suspended in 2 mL of ThinPrep buffer (PreservCyt; Hologic, Marlborough, MA) and vortexed for 2 minutes. Cervical samples were obtained by the provider using a rover brush and placed in ThinPrep buffer (PreservCyt) immediately after collection.

### Study Cohorts

Four separate general adult (aged ≥18 years) outpatient cohorts were recruited, each for different aspects of this study: 15 participants were recruited for analysis of swab diagnostic variability (Supplemental Figure S1), 144 participants were recruited for paired comparison of self-collected vaginal swab versus provider-collected cervical samples (Supplemental Figure S2), 68 participants were recruited for ambient stability studies (Supplemental Figure S3), and 78 participants were recruited for summer/winter temperature challenge studies (Supplemental Figure S4). For the self-collected to provider-collected comparison, all samples were collected from the colposcopy clinic; for all other studies, there was no enrichment for known positive samples (any HPV-positive results were incidental and indicative of a general population of volunteers).

### HPV Assay

Regardless of the collection source, HPV testing was performed using the Roche (Basel, Switzerland) Cobas 6800.^12^ The Cobas HPV test has three different channels that provide specific genotyping results for HPV16 [approximately limit of detection (LOD) mean cycle threshold (C_T_) = 35.0], HPV18 (approximately LOD mean C_T_ = 33.8), and other high-risk HPV (approximately LOD mean C_T_ = 32.4), which includes 12 pooled genotypes (HPV31, HPV33, HPV35, HPV39, HPV45, HPV51, HPV52, HPV56, HPV58, HPV59, HPV66, HPV68). In this article, samples are called co-positive if two of the three different channels detected HPV. The Cobas HPV test uses β-globin DNA as an internal control to confirm the presence and amplification of human DNA. HPV-negative/β-globin–positive results are reported negative; HPV-positive/β-globin–positive results are reported positive; and HPV-positive/β-globin–negative results are reported positive. Invalid results are specific to samples that are HPV negative/β-globin negative; samples with invalid results require recollection to complete the recommended screening. Assay/instrument validation and implementation were completed in accordance with College of American Pathologists standards in Clinical Laboratory Improvement Amendments–accredited laboratories. Briefly, manufacturer-supplied positive and negative controls were run 20 times within a single run and 20 times over 5 days to ensure instrument/assay precision was adequate. Additionally, accuracy was verified using residual discard known positive (*n* = 20 positive per channel; *n* = 60 total) and known negative (*n* = 20) provider-collected cervical samples in ThinPrep buffer. The HPV assay and instrument are US Food and Drug Administration approved for clinical use in HPV primary cervical cancer screening.

### Variation of Intra-User Self-Collected Vaginal Swab DNA Yields

Vaginal swabs for assessment of swab variability (*n* = 15) were obtained at the LetsGetChecked Laboratory (Monrovia, CA) by consenting individuals. Participants were provided five individually wrapped swabs and five empty collection transport tubes labeled 1 to 5. They were instructed to collect five vaginal swabs and to place the first swab into the tube labeled 1, and consecutively thereafter. Buffer was added to the swabs within 24 hours of collection, and they were evaluated for HPV (Supplemental Figure S1). C_T_ values for β-globin and/or HPV for the last swab (fifth swab) and first swab were used to calculate the ΔC_T_ between the sequential collections.

### Comparison of Provider and Self-Collected Samples for HPV Detection

Paired self-collected vaginal/provider-collected cervical samples (*n* = 144) were obtained from volunteers at their follow-up colposcopy visits and subsequently evaluated for HPV at the Kaiser Permanente Northern California Regional Reference Laboratory (Berkeley, CA) (Supplemental Figure S2). The self-collected dry vaginal swabs were suspended in buffer on receipt in the laboratory; the provider-collected cervical samples were placed in ThinPrep media immediately after collection. Paired collections were de-identified with pairs designated solely by study identification numbers and were sent to the laboratory in batches several times per month. Self-collected and provider-collected samples were batched together within the same analytical runs for HPV detection. C_T_ values for HPV and β-globin were compared between the two collections.

For the self-collected dry vaginal swabs, the number of days between collection and buffer suspension was recorded as a variable and was used for additional analysis to assess if extended time between collection and receipt at the laboratory would influence HPV detection. The difference in C_T_ value between the provider-collected cervical sample and the self-collected vaginal swab was calculated and plotted as a function of the number of days that the swab remained dry. Paired samples that amplified HPV or β-globin in only one of the collection sources were excluded from this subanalysis.

### Ambient 30-Day Stability of Dry Swabs

Participants (*n* = 68) self-collected two vaginal swabs (Supplemental Figure S3). The baseline sample was immediately suspended into ThinPrep buffer and evaluated for HPV within 24 hours of collection. The second swab remained dry for 4 to 41 days at uncontrolled ambient temperature indicative of the indoor laboratory environment. The change in amplification of β-globin and/or HPV as a function of the number of days the swab remained dry was calculated. Swabs that had an invalid result for the baseline sample (*n* = 3) were excluded from the stability experiment. Samples that were HPV positive but did not have β-globin amplification (*n* = 1) were included for HPV calculations only.

### Stability of Dry Swabs after Exposure to Temperatures Ranging from 22°C to 40°C or −10°C to 22°C over 72 Hours

Participants (*n* = 78) self-collected three vaginal swabs and placed them into empty collection tubes labeled day 0, summer simulated, and winter simulated (Supplemental Figure S4). Swabs labeled day 0 were suspended immediately into ThinPrep buffer and evaluated for HPV within 24 hours of collection (baseline). There was a variable number of days that the seasonally challenged swab remained dry before initiating the challenge.

Dry swabs were placed into a KB E4 environmental chamber calibrated to a National Institute of Standards and Technology standard thermometer that simulated highly specific temperature profiling. The profile is indicative of extremes a sample may endure during transit.

To simulate summer temperatures, samples cycled between 22°C and 40°C as follows: 40°C for 8 hours, 22°C for 4 hours, 40°C for 2 hours, 30°C for 36 hours, 40°C for 6 hours, and 22°C for 16 hours. Hereafter, this is referred to as the summer challenge.

To simulate winter temperatures, samples cycled between −10°C and 22°C as follows: −10°C for 8 hours, 18°C for 4 hours, −10°C for 2 hours, 10°C for 36 hours, −10°C for 6 hours, and 22°C for 16 hours. Hereafter, this is referred to as the winter challenge.

Following summer or winter challenge, the dry swabs were removed from the chamber and suspended in 2 mL of ThinPrep buffer before testing for HPV. C_T_ values between β-globin and/or HPV were compared between baseline, summer, and winter challenged samples. The difference in C_T_ value for the challenged swab from baseline was plotted as a function of the number of days the swab remained dry.

### Assessing Invalid Samples for Cellular Material

To better understand if invalid results from self-collected samples were due to an absence of cellular material during collection, a second internal cellularity control was challenged. A subset of self-collected vaginal swab samples (*n* = 20 invalid β-globin/HPV negative; *n* = 20 invalid β-globin/HPV positive; and *n* = 20 β-globin positive/HPV positive) were evaluated for the presence of RNase P to determine whether there was a difference in cellular recovery between samples that did and did not amplify the β-globin target. RNase P was amplified using the human RNase P Forward Primer/Probe Mix kit from IDT (Coralville, IA) and the Thermo Fisher Applied Biosystems 7500 Fast Dx Real Time PCR instrument (Waltham, MA).

### Statistical Analysis

Calculations and statistical analysis were completed using Google Sheets (Mountain View, CA), EP Evaluator version 12.3 (Data Innovations, Colchester, VT), and GraphPad Prism version 10.2.0 (GraphPad Software, Boston, MA).

## Results

### Variation of Intra-User Self-Collected Vaginal Swab DNA Yields

**Figure 1:**
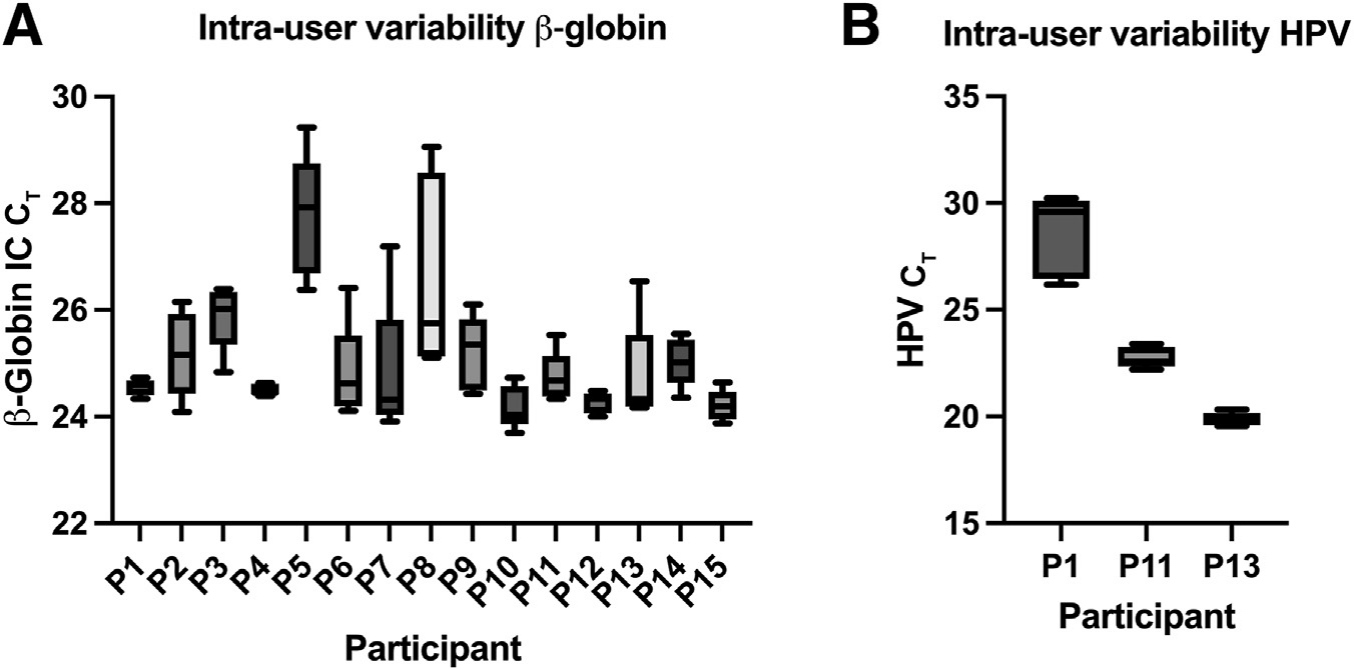
Box-and-whisker plots (minimum to maximum) of β-globin internal control (IC) amplification C_T_ for dry swab collection (**A**) and human papillomavirus (HPV) amplification C_T_ across five consecutively collected swabs for the subset of participants (Ps) who were HPV positive (P1, HPV 16; P11 and P13, HPV other; **B**). *n* = 15 participants collecting five consecutive swabs each (**A**); *n* = 3 (**B**).

The diagnostic variability of self-collected vaginal swabs was assessed by comparing testing results from five swabs collected sequentially from study participants (*n* = 15) (Supplemental Figure S1). The average CV for the β-globin internal control C_T_ per individual across the five collections was 2.7% (range, 0.4% to 6.9%) (Figure 1A). The average difference between β-globin C_T_ values for the fifth swab collected and the first swab collected was −0.1 (SD = 1.5; range, −2.9 to 4.0). There was no statistically significant difference in β-globin amplification between the first and last swabs collected (*P* = 0.724, paired two-tailed *t*-test). For the subset of specimens testing positive for HPV (*n* = 1 HPV16; *n* = 2 HPV other) (Figure 1B), there was 100% qualitative concordance across the five collections. Quantitatively, there was an intra-individual CV of 6.8%, 2.1%, and 1.6% for HPV 16 C_T_ and the two HPV other genotypes. The average difference between the HPV C_T_ value for the fifth swab collected and the first swab collected was 0.2 (C_T_ difference of −0.4 for HPV16 and −0.3 and 0.1 for HPV other), which was not statistically significant (*P* = 0.293, paired two-tailed *t*-test). These data suggest that there is minimal intrapatient diagnostic variability for self-collected vaginal swabs, and that for analytical studies, multiple swabs collected from the same participant will have minimal baseline differences.

### Comparison of Self-Collected Vaginal and Provider-Collected Cervical Samples

**Figure 2:**
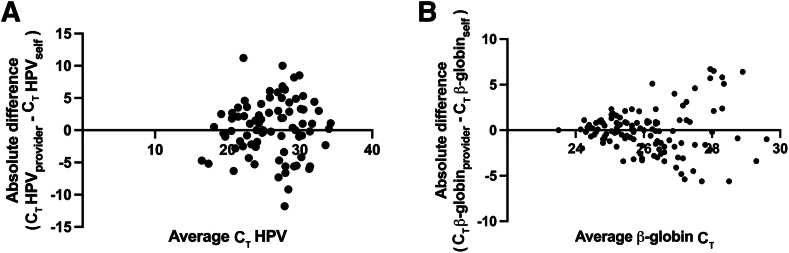
Bland-Altman plots illustrating the C_T_ difference for human papillomavirus (HPV; **A**) and β-globin internal control (**B**) between the self-collected vaginal swabs and provider-collected cervical samples.

Paired, self-collected vaginal swab and provider-collected cervical samples (*n* = 144) were evaluated for HPV molecular testing, and the results were compared (Supplemental Figure S2). Amplification of HPV and the β-globin internal control was equivalent between the two collections (*P* = 0.454 for HPV and *P* = 0.835 for β-globin, paired two-sided *t*-test) (Figure 2).

For a total of 127 participants (88%), test result interpretations were fully concordant with a positive percentage agreement of 84.2% for HPV detection between collection modalities (Supplemental Table S1). In total, 64 HPV other, 22 HPV16, and 9 HPV18 infections were identified using one or both collection types. In 59 participants, concordant HPV-negative testing was observed between the two collection types; three of these samples did not amplify β-globin in the self-collected sample, giving invalid results, and therefore would have required a follow-up collection to confirm the negative HPV result. In 68 participants, HPV was concordantly identified between different collections (*n* = 19 HPV16, *n* = 8 HPV18, and *n* = 53 HPV other). This group included 59 samples that were positive for HPV16, HPV18, or HPV other, and 9 samples having more than a single positive result (ie, co-positive or HPV genotypes detected in more than one of the assay channels). Additionally, there were two partially concordant samples (sample identifiers 136 and 109) (Table 1), the first of which was co-positive on the self-collected sample (HPV16 and other), but for which only a single channel (HPV16) was positive from the provider-collected sample; the second was co-positive on three channels for the self-collected sample (HPV16, HPV18, and HPV other) but with only two identified from the provider-collected sample (HPV18 and HPV other). Clinical triage of these participants would not change based on this discrepancy because HPV16 or HPV18 was detected in both collections.

**Table 1:** Samples Discrepant for HPV Detection between Self-Collected Vaginal and Provider-Collected Cervical Samples

Sample ID	Time the self-collected swab remained dry, days	Cervical HPV C_T_ (when positive)	Vaginal HPV C_T_ (when positive)	Cervical β-globin C_T_	Vaginal β-globin C_T_	Strain
**2**	3	Neg	29.9	25.8	25.6	Other
**13**	30	Neg	34.4	26	26.7	Other
**76**	1	Neg	28.5	25.5	24.1	16
**76**	1	Neg	30.5	25.5	24.1	18
**87**	21	Neg	34.3	25.3	No amplification	Other
**109**∗	28	Neg	37.4	27.1	No amplification	16
**111**	9	Neg	31	24.4	24.2	Other
**129**	14	Neg	30.9	31.3	25.7	Other
**136**∗	22	Neg	29.6	24.2	25.9	Other
**138**	21	Neg	34.4	Invalid	31.3	Other
97	Not available	31.5	Neg	24.6	26	Other
112	22	29.9	Neg	25.6	26.6	16
16	28	26	Neg	26.9	27.8	Other
89	15	30.3	Neg	25.4	31.2	Other
42	22	29.7	Neg	25.5	Invalid	Other
10	30	Neg	Neg	25.9	Invalid	ND
12	30	Neg	Neg	25.7	Invalid	ND
19	22	Neg	Neg	24.4	Invalid	ND

The first 10 rows (boldfaced sample IDs) were HPV positive by self-collect and HPV negative by provider collect. The middle 5 rows (underlined sample IDs) were HPV negative by self-collect and HPV positive by provider collect. The last 3 rows (normal font sample IDs) were invalid for β-globin for the self-collected and HPV negative for the provider-collected samples.

HPV, human papillomavirus; ID, identifier; Neg, negative.

∗ Samples 109 and 136 were tripositive and co-positive by self-collect and were co-positive and single strain positive by provider collect, respectively.

There were 15 participants in whom discrepant testing results occurred between the provider and self-collected samples (Table 1). These discrepancies grouped into three general categories.

The first category comprised seven unique patients who were positive for HPV (*n* = 6 HPV other, *n* = 1 HPV16, and *n* = 1 HPV18) by self-collected vaginal swab, but who were negative for HPV on the provider-collected cervical samples; one of these self-collected samples was co-positive for HPV16 and HPV other but was negative for all HPV channels by provider collect. β-Globin amplified similarly in both collections (C_T_ = 24.1 for self and 25.5 for provider; *P* = 0.296, paired two-tailed *t*-test).

The second category of discrepancy was composed of samples from five participants who were negative for HPV by self-collection but positive by provider collect (*n* = 4 HPV other, and *n* = 1 HPV 16). The range of C_T_ values for HPV on the provider collect was 26.0 to 31.5 (mean C_T_ = 29.5; SD = 2.1). For these samples, four had valid amplification of β-globin by both methods (average C_T_ = 25.6 and SD = 1.0 for provider collect; average C_T_ = 27.9 and SD = 2.3 for self-collect), and one sample was invalid for β-globin amplification and HPV negative by self-collect, being compatible with sample insufficiency, but was HPV other positive by the provider-collected sample.

The third category of discrepancy comprised three samples that were partially invalid; results were HPV negative for both self-collected and provider-collected samples, but only yielded successful amplification of β-globin on the provider-collected samples. The self-collected pair to these cervical samples had been stored as dry swabs for 22 days (*n* = 1) and 30 days (*n* = 2). Lack of β-globin amplification without HPV detection is considered incomplete for screening and, therefore, in these cases the self-collected participants would require additional sampling, but otherwise clinical interpretation would not be affected.

In summary, of the 144 participants, HPV was detected 85 times in participants using clinician-collected samples and 90 times in participants using self-collected samples.

**Figure 3:**
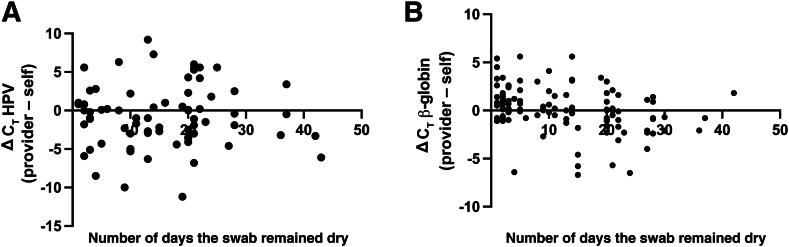
The difference in the C_T_ values between the provider-collected cervical and the self-collected vaginal samples is plot as a function of the number of days the swab remained dry. The ΔC_T_ values for the human papillomavirus (HPV; **A**) and β-globin internal control (**B**) are shown. Positive values indicate that there was more DNA amplified from the self-collected sample; negative values indicate there was more DNA amplified on the provider-collected sample.

For the concordant HPV-positive collections, and for β-globin across all pairs, there was no difference in C_T_ value between collections (Figure 2). We, therefore, performed a subanalysis to see if the number of days the self-collected vaginal swab remained dry influenced DNA amplification (Figure 3). After excluding samples where collection date was missing (*n* = 12 samples, *n* = 10 HPV positive) or where β-globin failed to amplify in one of the collections (*n* = 17), the authors evaluated relative amplification of β-globin (*n* = 115) and HPV (*n* = 71) between provider and self-collected samples as a function of the number of days the swab remained dry (Figure 3). The average amount of time these swabs remained dry was 13.7 days (SD = 10.3 days). For concordant positive (*n* = 71) and concordant negative (*n* = 44) samples, the average amount of time the swab remained dry was 14.6 (SD = 10.1) and 11.2 (SD = 9.9) days, respectively. For discrepant samples where self-collect was HPV positive and provider-collect negative (*n* = 10), the average amount of time the swab remained dry was 16.6 days (SD = 10.4 days) (*P* = 0.09, unpaired two-tailed *t*-test for number of days false negative provider remained dry versus number of days for all concordant samples remained dry); discrepant samples where self-collect was HPV negative and provider-collect positive (*n* = 5), the average time the swab remained dry was 21.8 days (SD = 5.3 days) (*P* = 0.31, unpaired two-tailed *t*-test for number of days false negative self-collected remained dry versus number of days concordant samples remained dry). For samples that were invalid by self-collect and HPV negative by provider collect (*n* = 3), the average amount of time the swab remained dry was 27.3 days (SD = 4.6 days) (*P* = 0.02, unpaired two-tailed *t*-test for number of days for three invalid samples versus number of days for all concordant samples). There was no relationship between the number of storage days and change in HPV C_T_ (*R* = 0.0012; *P* = 0.991), although there was a significant decrease in β-globin amplification over time (*R* = 0.3124; *P* = 0.0005).

### Ambient 30-Day Stability of Dry Swabs

The stability of vaginal swabs when stored at ambient temperatures for ≥30 days was evaluated (*n* = 65) (Supplemental Figure S3).

**Figure 4:**
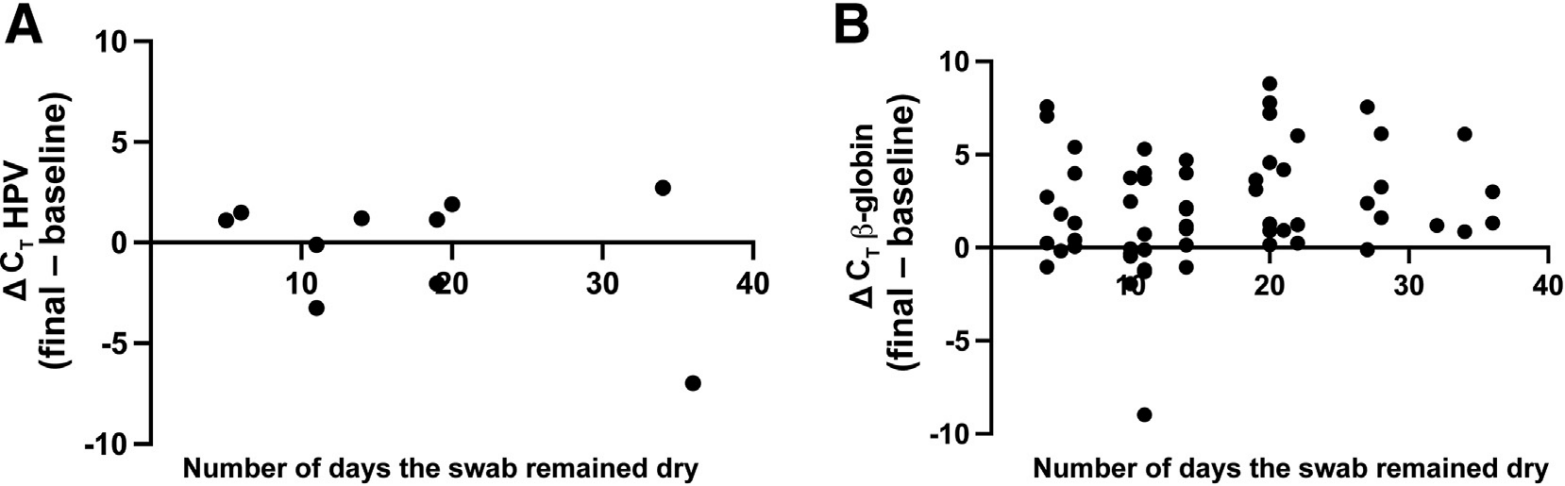
The difference in the C_T_ values between the extended room temperature and baseline self-collected vaginal sample is plot as a function of the number of days the second swab remained dry. The ΔC_T_ values for the human papillomavirus (HPV; **A**) and β-globin internal control (**B**) are shown. Positive values indicate that there was more DNA amplified from the baseline swab.

The average C_T_ for β-globin in the initial (baseline) samples, tested within 24 hours of collection, was 26.2 (SD = 2.0). Swabs remained dry for an average of 16.4 days (SD = 9.5 days). After suspending in buffer, six swabs were considered invalid based on negative β-globin amplification. For swabs that amplified β-globin (*n* = 59), the average β-globin C_T_ value was 26.4 (SD = 2.5) for initial swabs and 28.5 (SD = 3.2) for stored swabs (*P* ≤ 0.0001, paired two-sided *t*-test). The change in C_T_ for β-globin between the extended room temperature sample as a function of the number of days was significant and is shown in Figure 4B (*R* = 0.187, *P* < 0.0001). There were 11 HPV-positive samples present in the baseline (*n* = 8, 1, and 2 for HPV other, HPV16, and HPV18, respectively). The average baseline HPV C_T_ for these samples at baseline was 29.0 (SD = 3.3). After storage at room temperature, 10 samples remained HPV positive (average C_T_ = 28.5; SD = 5.4; positive percentage agreement = 91.0%), and their C_T_ was not significantly different from baseline readings (*P* = 0.491, two-tailed paired two-sided *t*-test). For the one sample where HPV other was not detected after storage, the baseline HPV other C_T_ was near the LOD (31.0) and the swab was stored for 28 days. There was no relationship between the number of storage days and change in HPV C_T_ (*R* = –0.332; *P* = 0.349) (Figure 4A).

These data suggest that HPV detection is unlikely to be compromised when the dry swab is stored at ambient temperatures for at least 30 days and that β-globin is a sensitive indicator of sample integrity.

### Stability of Dry Swabs after Exposure to Temperatures Ranging from 22°C to 40°C or −10°C to 22°C over 72 Hours

Sample stability (*n* = 78 participants collecting three swabs each) was assessed next under controlled environmental conditions intended to simulate summer and winter temperature extremes (Supplemental Figure S4). There were five samples having invalid β-globin at the baseline measurement and were therefore excluded from further challenges, leaving 73 dry specimen pairs, one of the pair used for winter and the other for summer challenges.

**Figure 5:**
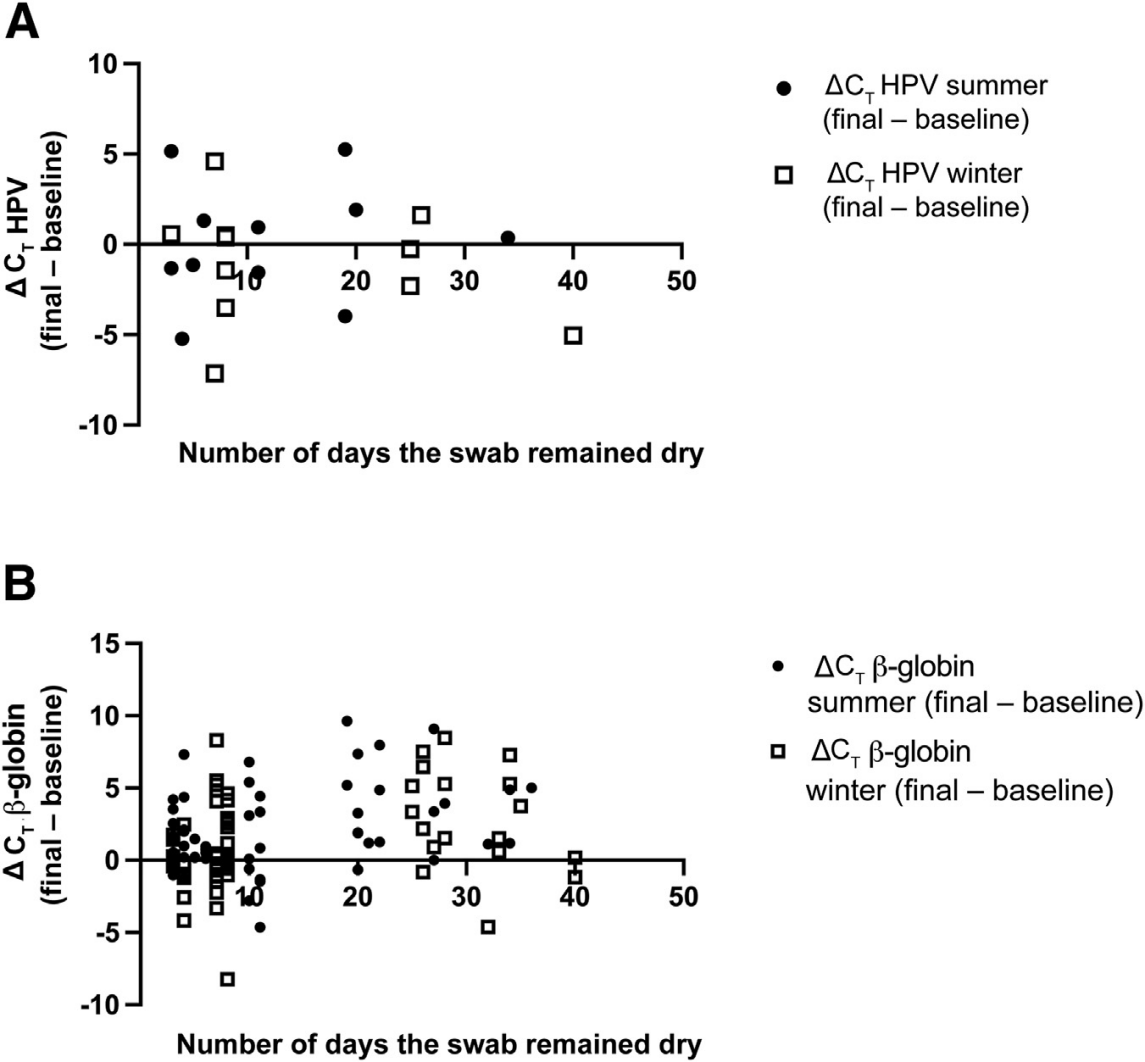
The difference in the C_T_ values between the summer (closed circles) or winter (hollow squares) and baseline self-collected vaginal sample is plot as a function of the number of days the second swab remained dry. The ΔC_T_ values for human papillomavirus (HPV; **A**) and β-globin internal control (**B**) are shown. Positive values indicate that there was more DNA amplified from the baseline swab.

After exposure to the summer challenge, an additional 13 samples were invalid (baseline β-globin C_T_ for these samples had an average value of 27.1 and SD = 3.2). For samples where there was β-globin amplification for both baseline and summer challenged (*n* = 59), the average β-globin C_T_ value was 26.2 (SD = 2.3) and 28.2 (SD = 3.2) for the baseline and summer challenged pairs, respectively. Comparing paired β-globin C_T_ before and after summer challenge was statistically significant (*P* ≤ 0.0001, paired two-sided *t*-test) (Figure 5B). β-Globin C_T_ was also influenced by the number of days the swab remained dry (*R* = 0.376, *P* = 0.004).

There were 14 HPV infections detected at baseline. Following summer challenge, 13 remained detectable and an additional infection was detected that was not positive for the baseline sample. The paired difference in C_T_ value between the samples that were positive for HPV on baseline and after summer challenge was not significant (*P* = 0.316) (Figure 5A). The sample that was positive on baseline, but negative following summer challenge, had a C_T_ value near the LOD for HPV other (31.0). The sample that was positive after summer challenge, but negative at baseline, had a near the LOD for HPV18 (37.1). There was no relationship between the number of storage days and change in HPV C_T_ (*R* = −0.242; *P* = 0.426).

A separate set of specimens with valid β-globin amplification for the baseline swab (*n* = 74) were challenged with extreme winter temperatures, after which six samples were considered invalid and subsequently excluded (baseline β-globin C_T_ for these samples had an average value of 26.3 and SD = 1.7). For samples where there was β-globin amplification on both baseline and winter challenged (*n* = 69), the average C_T_ value was 26.3 (SD = 2.5) and 27.6 (SD = 3.2) for the baseline and winter challenged samples, respectively (Figure 5B). Comparing paired β-globin C_T_ before and after winter thermal cycling was statistically significant (*P* = 0.0024, paired two-sided *t*-test). β-Globin C_T_ was also influenced by the number of days the swab remained dry (*R* = 0.2691, *P* = 0.03). There were 14 HPV infections detected at baseline. Following winter challenge, 13 remained detectable and an additional 2 infections were detected that were not positive for the baseline sample. The paired difference in C_T_ value between the samples that were positive for HPV on baseline and after winter challenge was not significant (*P* = 0.785) (Figure 5A). The sample that was positive on baseline, but negative following winter challenge, had a C_T_ value for 31.0 for HPV other. The samples that were positive after winter challenge, but negative at baseline, had a C_T_ = 35.7 (HPV 18) and 35.3 (HPV 16). All HPV C_T_ values for these discrepancies were near the LOD for the genotypes relevant to the specific HPV channel. There was no relationship between the number of storage days and change in HPV C_T_ (*R* = −0.3303, *P* = 0.271).

Combined, these data suggest that peak summer and winter conditions may influence β-globin amplification but are unlikely to influence HPV detection.

### RNase P Detection

**Figure 6:**
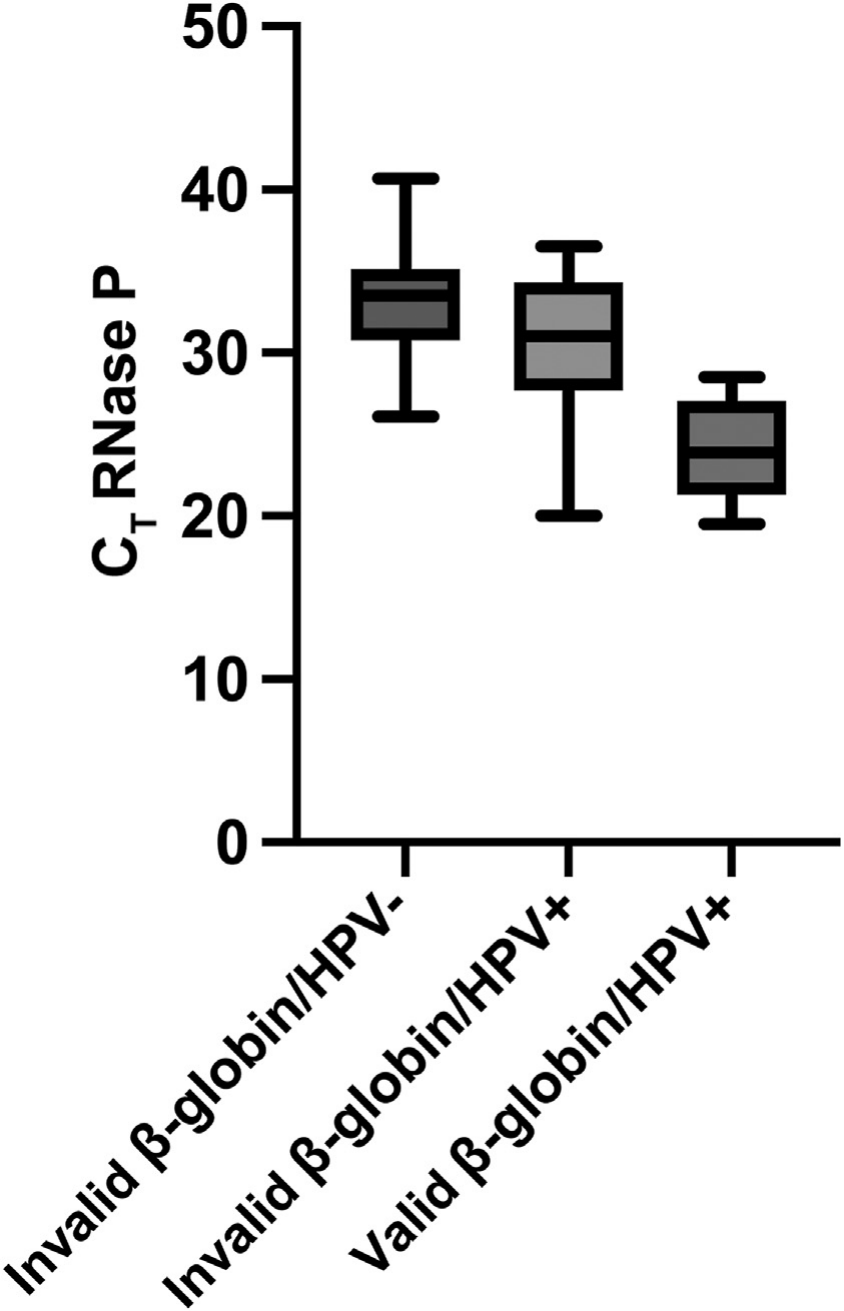
Box-and-whisker plots (minimum to maximum) of RNase P amplification in samples with and without human papillomavirus (HPV) amplification.

RNase P is a commonly used housekeeping gene for swab-collected viral assays used to confirm adequate collection and amplification.^13^ The authors therefore orthologously assessed RNase P amplification in a subset of specimens as an independent measurement of DNA content. RNase P was detectable in 12 of 20, 19 of 20, and 20 of 20 samples that were invalid for β-globin/negative for HPV, invalid for β-globin/positive for HPV, and valid for β-globin/positive for HPV, respectively. Samples that had valid β-globin amplification had a significantly higher concentration of RNase P (average C_T_ = 23.9; SD = 2.9) compared with those where β-globin did not amplify (*P* < 0.0001 for both cohorts regardless if HPV was detected) (Figure 6). Samples that were HPV positive, but did not amplify β-globin, had an average of RNase P C_T_ 30.2 (SD = 4.6), whereas the samples that were invalid and did not amplify β-globin or HPV had an average of RNase P C_T_ of 33.2 (SD = 3.6). There was no significant difference in RNase P C_T_ between samples that were HPV negative and HPV positive without β-globin amplification (*P* = 0.067, unpaired two-sided *t*-test). The authors conclude that lack of amplification for β-globin from self-collected specimens was consistent with low cellularity.

## Discussion

The Roche Cobas HPV assay has been indicated for self-collected specimens in the European Union, where screening programs using at-home collection have been successful for their target population.^14^ Similar screening strategies have had positive results in Japan.^15^ The implementation of these programs has measurably improved access for individuals in rural communities and those with limited access to health care centers during operating hours. Indeed, recent pilot studies in the United States showed significant improvement in HPV screening for low-income and underscreened populations when remote self-collection was offered.^16^^,^^17^ An at-home self-collection can also reduce discomfort and stress for some patients.^18^, ^19^, ^20^ Despite the benefits, it is necessary to ensure that any significant potential quality differences between a provider-collected and home-based self-collected sample are addressed. Although multiple studies have reported clinical equivalence between these collection types,^21^ there are little data in the literature that provide clinical laboratories guidance to validate self-collected samples. A recent publication reviewing the implementation challenges to national HPV screening programs in Australia highlighted a lack of laboratory readiness to accept self-collected specimens as an unexpected barrier.^22^

Here, we provide detailed quantitative data to define variability in testing results between provider-collected and self-collected samples. Our results were highly concordant between the different collections (total agreement = 90.3%; positive percentage agreement = 84.2%), showing equivalency of testing results between the two modalities. Among discordant samples, cases in which the self-collected sample detected HPV and the clinician-collected sample was negative outnumbered cases in which the converse was true. In addition to providing encouraging support for the use of home-collected specimens, this study can offer individual laboratories a template for analogous validations in their own practice, which are a baseline requirement for implementation.

Dry swabs are considered an exempt human specimen, meaning that dry swabs are not considered at-risk biological material, enabling their transport to a central laboratory via standard mail. However, successful remote collection requires HPV DNA to be stable during transit to preserve integrity of the specimen for subsequent testing. We exposed self-collected dry swabs to extreme temperatures that exceed those of peak winter/summer months. It was found that these temperatures can lead to degradation for the human β-globin internal control, but the HPV DNA target is largely unaffected. The cumulative result of these effects is a greater likelihood of an invalid result for self-collected samples during inclement seasons, but importantly, without impacting the negative predictive value of the testing pathway. Real-world data indicate that the true invalid rate for samples is minimal [6/1206 samples invalid in Self-Testing Options in the Era of Primary HPV Screening for Cervical Cancer Trial (STEP) trial],^10^ and indeed, orthologous testing of insufficient specimens in this study by RNase P real-time quantitative PCR suggests that self-collected specimens considered invalid were a result of poor, but not absent, cellularity. Overall, these data support that self-collected HPV samples have adequate stability for transit commensurate with US Postal Service first-class mail. Furthermore, they indicate that stability can be defined by the presence or absence of β-globin amplification.

There are several limitations to this study. Dry swabs are an unconventional sample type, and therefore there are no commercially available matrix-matched quality control specimens. For this assay, the presence of an internal control mitigates the need for such, but nevertheless monitoring matrix-matched performance is preferable. Similarly, with this sample type, it is not possible to test sample stability longitudinally because it is a single unique dry swab and therefore the stability experiments were performed using multiple collections that may have had differing baselines. However, the interuse variability experiments illustrated that the difference between multiple swabs collected by the same individual will only have minimal variation. For some validation analyses, there were a limited number of HPV-positive collections, which limits statistical power. A small subset of specimens was invalid by one of the two collections, preventing mathematical comparison of amplification. Although the results are likely generalizable, this study was designed to test performance for a particular platform (Roche Cobas) and specific, actionable HPV genotypes. Lastly, a human factors assessment (usability study) of the collection process was not directly performed. This was assessed indirectly because all participants were untrained volunteers whose samples were collected unsupervised. Poor collections result in an increased invalid rate (no amplification of β-globin), which would require repeat collection and not lead to reporting of erroneous results. Future work should focus on improving collection robustness, as clinically warranted and realistic. Regardless of who is collecting the sample, invalid collection rates should be monitored and quality assurance thresholds should be set by the laboratory.

In conclusion, we provide a robust quantitative analysis for amplification of HPV from self-collected dry vaginal swabs relative to provider-collected cervical samples and evaluated the effects of ambient storage length and temperature challenges on testing outcomes. This work provides further support that HPV screening can be performed with high accuracy on vaginal swabs that are self-collected remotely from the testing site. Although home-collected samples cannot replace the need for health care provider encounters, their use should facilitate more efficient clinical encounters by providing HPV testing results in advance.

## Disclosure Statement

At the time of experimentation and/or writing, M.Q., A.R.N., M.R., and D.N.G. were salaried employees at LetsGetChecked.
