# Supplementary material for: Evaluation of Pre-Analytical Variables for Human Papillomavirus Primary Screening from Self-Collected Vaginal Swabs
Source: J Mol Diagn. 2024 Jun;26(6):487–97. doi: 10.1016/j.jmoldx.2024.02.006 (PMC11238274; doi:10.1016/j.jmoldx.2024.02.006)
Supplement: Supplemental Table S1 [file mmc1.docx]

**Supplemental Table 1**: HPV agreement for self-collected vaginal (S) and provider collected cervical samples (P).

| **HPV Genotype** | **P-/S-** | **P+/S-** | **P-/S+** | **P+S+** | **Total % agreement** | **Positive % agreement** |
| --- | --- | --- | --- | --- | --- | --- |
| Any | 59 | 5 | 10 | 80 | 90.3 | 84.2 |
| HPV16 | 122 | 1 | 2 | 19 | 97.9 | 86.4 |
| HPV18 | 135 | 0 | 1 | 8 | 99.3 | 88.9 |
| HPV other | 80 | 4 | 7 | 53 | 92.4 | 82.8 |
